# 855 Streamlining Burn Care: A Comprehensive Algorithm for Lower Extremity Burn Management

**DOI:** 10.1093/jbcr/iraf019.386

**Published:** 2025-04-01

**Authors:** Mare Kaulakis, Alexis Henderson, Christopher Fedor, Hilary Liu, José Arellano, Garth Elias, Alain Corcos, Jenny Ziembicki, Francesco Egro

**Affiliations:** University of Pittsburgh School of Medicine; University of Pittsburgh School of Medicine; University of Pittsburgh School of Medicine; University of Pittsburgh Medical Center; University of Pittsburgh Medical Center; University of Pittsburgh Medical Center; University of Pittsburgh Medical Center; University of Pittsburgh Medical Center; University of Pittsburgh Medical Center

## Abstract

**Introduction:**

Lower extremity burns pose significant challenges, often leading to morbidity and long-term functional impairment. Despite advancements in burn care, treatment variability leads to differences in healing, amputation rates, and recovery. This study aims to develop a standardized treatment algorithm based on evidence-based practices to improve outcomes in lower extremity burn care.

**Methods:**

A retrospective analysis was conducted on patients presenting at a single ABA-verified burn center from 2012-2023 with lower extremity burns. Data collected included demographics, burn characteristics, treatment strategies, and outcomes. Logistic regression analyses were performed to evaluate associations between burn factors and likelihood of surgical intervention.

**Results:**

The study included 558 patients (64.87% male, 35.13% female), with a mean age of 38.62 ± 24.25 years and a mean BMI of 26.64 ± 8.12. Most burns were caused by flame (48.57%; n=271) and scald (34.59%; n=193), followed by chemical (3.23%; n=18) and electrical (1.26%; n=7), with a mean total body surface area (TBSA) of 10.51 ± 14.41 and lower extremity BSA of 3.06 ± 0.85. Full thickness (35.66%; n=199) and deep partial thickness (34.95%; n=195) burns were most common.

Of the 300 patients (53.76%) who underwent surgical intervention, 13 (4.33%) had superficial partial thickness burns, 111 (37%) had deep partial thickness burns, and 176 (58.67%) had full thickness burns to the lower extremities. Common procedures included excision plus autograft (43.33%; n=130), and a two-stage operation with cadaver graft followed by autograft (36%; n=108). Each 1% increase in TBSA was associated with a 4% higher likelihood of needing surgery (OR=1.04, p=0.0115), a 5% increase in requiring excision plus cadaveric allograft (OR = 1.05, p=0.0027), and a 6% decrease in the odds of needing excision plus autograft (OR = 0.94, p=0.0025). Patients with deep partial thickness burns were 13.23 (OR = 13.23, p=0.000) times more likely to undergo surgical intervention than those with superficial burns, while patients with full thickness burns had a 64.55 (OR = 64.55, p=0.0000) times higher likelihood of requiring surgery.

Ultimately, 15.42% (n=86) of all patients had hypertrophic scarring, 29.03% (n=162) developed cellulitis, and 21.67% (n=65) of surgical patients experienced graft loss.

**Conclusions:**

A standardized treatment algorithm for lower extremity burns is crucial for improving outcomes. This study shows TBSA and burn depth significantly influence surgical decisions, with larger and deeper burns often requiring excision and cadaveric allograft, followed by autograft, due to patient instability or limited healthy skin.

**Applicability of Research to Practice:**

This study highlights the need for a standardized treatment algorithm to guide clinical decisions, streamline protocols, and improve outcomes in lower extremity burn care.

**Funding for the Study:**

N/A

